# Health Concerns for LGBTQ+ Caregivers of People With Alzheimer’s Disease and Related Dementias

**DOI:** 10.1093/geroni/igab046.1563

**Published:** 2021-12-17

**Authors:** Krystal Kittle, Joel Anderson, Jennifer Pharr, Sheniz Moonie, Jason Flatt

**Affiliations:** 1 University of Nevada, Las Vegas, Las Vegas, Nevada, United States; 2 University of Tennessee at Knoxville, Knoxville, Tennessee, United States; 3 University of Nevada Las Vegas, Las Vegas, Nevada, United States

## Abstract

We examined four states with data on LGBTQ+ identity and the ADRD caregiving modules from the 2019 Behavioral Risk Factor Surveillance System. Multivariable regression models examined the associations between LGBTQ+ identity and health outcomes. Among the ADRD caregivers, 55,920 (4.7%) identified as LGBTQ+. Compared with non-LGBTQ+ caregivers, LGBTQ+ caregivers were younger and more likely to live in rural counties. Half of LGBTQ+ caregivers spent 20+ hours per week providing care, and nearly 72% reported helping with personal care. LGBTQ+ caregivers reported more days when their mental health was not good than non-LGBTQ+ caregivers (B = 8.01;95% CI= 2.32-13.75). Female caregivers overall were twice as likely than males to experience depression (OR = 2.11;95% CI=1.29-3.45). These findings provide insight into characteristics of LGBTQ+ caregivers and their health concerns. Interventions that promote mental health and reach diverse LGBTQ+ caregivers in rural communities are crucial in supporting LGBTQ+ caregivers of people with ADRD.

